# Low expression of miR-27b in serum exosomes of non-small cell lung cancer facilitates its progression by affecting EGFR

**DOI:** 10.1515/med-2022-0472

**Published:** 2022-04-27

**Authors:** Xiying Cao, Weixiang Zhong, Shaoming Guo, Zuxiong Zhang, Chunfa Xie

**Affiliations:** Department of Thoracic Surgery, The First Affiliated Hospital of Gannan Medical University, Economic Development District, Ganzhou, 341000, China; Department of Thoracic Surgery, The First Affiliated Hospital of Gannan Medical University, No. 128 Jinling Road, Economic Development District, Ganzhou, 341000, China

**Keywords:** miR-27b, non-small cell lung cancer, exosome, EGFR, ROC curve, Pearson analysis, CA125, CEA, CYFR21-1

## Abstract

Non-small cell lung cancer (NSCLC) is a malignant tumor. Serum exosomal miR-27b is related to tumor diagnosis. We explored the roles of serum exosomal miR-27b in NSCLC. NSCLC patients were assigned to NSCLC-early/terminal groups, with healthy subjects as controls. miR-27b expression was assessed using reverse transcription-quantitative polymerase chain reaction, and its diagnostic efficiency was analyzed using the receiver operating characteristic curve. The correlation between serum exosomal miR-27b expression and tumor markers carcinoembryonic antigen 125 (CA125), carcinoembryonic antigen (CEA), and cytokeratin 19-soluble fragment (CYFRA21-1) was analyzed using the Pearson analysis. The downstream target genes were predicted. Epidermal growth factor receptor (EGFR) level was assessed using enzyme-linked immunosorbent assay. Correlations of miR-27b expression with serum EGFR level and CA125, CEA, and CYFRA21-1 levels were analyzed using the Pearson analysis. Serum exosomal miR-27b was diminished in NSCLC and was further decreased in the NSCLS-terminal group. The sensitivity of miR-27b < 0.8150 for NSCLC diagnosis was 76.64%, and the specificity was 83.33%. Serum exosomal miR-27b was negatively correlated with CA125, CEA, and CYFRA21-1. miR-27b targeted EGFR. Serum EGFR was raised in NSCLC and was further elevated in the NSCLS-terminal group. miR-27b expression was negatively correlated with EGFR level. EGFR level was positively correlated with CA125, CEA, and CYFRA21-1 levels. Collectively, low expression of miR-27b assisted NSCLC diagnosis, and miR-27b exerted effects on NSCLC through EGFR.

## 1 Introduction

Lung cancer accounts for most cancer-related mortalities all over the world [1]. Approximately 85% of patients with lung cancer have histological subtypes collectively identified as non-small cell lung cancer (NSCLC) [2]. The risk factors for NSCLC include cigarette smoking, unhealthy diet, alcohol, occupational exposure to carcinogens, air pollution, and lack of physical activity [3]. Because of rapid metastasis, high recurrence rate, and the lack of efficient diagnostic biomarkers, the survival rate and prognosis of NSCLC remain poor [4]. The symptoms of NSCLC are not obvious, and as a result, most patients are in the middle and advanced stage when they are diagnosed, and the 5-year survival rate of patients with advanced NSCLC is less than 20%, while that of the patients with early NSCLC can reach 80% [5]. Therefore, early identification without obvious clinical symptoms can improve the prognosis [6].

Exosomes are membrane-bound extracellular vesicles released by cells, with a size of 40–150 nm and composed of double lipid membranes, which can promote intercellular communication [7]. Exosomes derived from the host cells can be absorbed by the distant or adjacent cells and exert their biological effects on these receptor cells, which have been implicated in disease occurrence and development, including cancer [8]. Exosomes show clinical potential as the biomarkers and therapeutics in NSCLC [9]. microRNAs (miRNAs) are a kind of non-coding small RNAs, which can regulate the post-transcriptional expressions of genes and play an imperative role in cell growth, metabolism, differentiation, and apoptosis [10]. miRNAs are identified to be the biomarkers for a variety of cancers [11]. For instance, miR-17-5p can be used as a biomarker in breast cancer, miR-135b-5p can be utilized as a diagnostic standard for colorectal cancer, and miR-485-5p can be applied as a potential biomarker in colorectal cancer [12,13,14]. Exosomes contain many biological molecules such as miRNAs. Due to the protection of lipid bilayer, exosomal miRNAs have higher stability than serum miRNAs. Therefore, it is worth exploring whether serum exosomal miRNAs are more valuable for the early diagnosis of NSCLC and other malignant tumors. It has been reported that exosomal miRNAs may be effective and promising biomarkers for the early diagnosis of NSCLC [15]. miR-27b represses epithelial–mesenchymal transition and chemoresistance in NSCLC by targeting Snail1 [16]. Epidermal growth factor receptor (EGFR) is a kind of transmembrane protein with the activity of cytoplasmic kinase that transduces essential growth factor signaling from extracellular milieu to cells and is expressed in over 60% of NSCLC cells [17]. However, there is little research on the clinical value of serum exosomal miR-27b in NSCLC and whether miR-27b affects NSCLC progression through EGFR. This study assessed the expression patterns and diagnostic value of serum exosomal miR-27b in NSCLC, to offer a certain reference value for NSCLC diagnosis.

## 2 Materials and methods

### 2.1 Ethics statement

The experiments were authorized by the academic ethics committee of the First Affiliated Hospital of Gannan Medical University. All subjects involved were fully informed of the objective of the study and signed informed consent before sampling.

### 2.2 Subjects and grouping

Totally, 137 NSCLC patients (80 males and 57 females, at the average age of 61.0 ± 5.4 years) diagnosed from November 2018 to March 2021 in the First Affiliated Hospital of Gannan Medical University were selected as the subjects. The exclusion criteria were as follows: (1) a history of lung surgery; (2) a history of cancer, radiotherapy, and chemotherapy; (3) complicated with immune deficiency diseases; and (4) severe impairment of heart, liver, and kidney function. All patients were diagnosed with NSCLC by histopathological examination, including 31 cases of lung adenocarcinoma, 99 cases of lung squamous cell carcinoma, and 3 cases of large cell carcinoma according to the 2015 World Health Organization Classification of Lung Tumors, and 64 cases of I–III A stage and 73 cases of III–IV B stage according to the eighth edition of the tumor, node and metastasis classification of lung cancer published by the 2009 International Association for the Study of Lung Cancer. Simultaneously, 60 healthy subjects who accepted physical examination in the First Affiliated Hospital of Gannan Medical University were selected as the control group, including 35 males and 25 females with an average age of 59.4 ± 6.8.

### 2.3 Data collection

At admission, the basic clinical information of all patients, including age, gender, and smoking history, was recorded. The peripheral blood of all subjects was collected under fasting conditions in the morning. After 24 h, the samples were centrifuged with the supernatant collected and stored at −80°C. Hemolysis was avoided in the whole process. The expression of serum exosomal miR-27b was detected using reverse transcription-quantitative polymerase chain reaction (RT-qPCR); the serum level of EGFR was examined using the enzyme-linked immunosorbent assay (ELISA) method; the alkaline phosphatase (ALP) level was examined by the enzyme method using a biochemical analyzer (AU2700; Olympus, Tokyo, Japan); the levels of hemoglobin and serum calcium were assessed using the colorimetry method; and the serum levels of carcinoembryonic antigen 125 (CA125), carcinoembryonic antigen (CEA), and cytokeratin 19-soluble fragment (CYFRA21-1) were examined using the electrochemiluminescence immunoassay method.

### 2.4 Extraction and identification of serum exosomes

The exosomes were extracted using the serum exosome extraction kit (Norgen Biotek, Ontario, Canada) in strict accordance with the instructions. The 10 µL serum exosome sample was placed on a copper mesh for 1 min, and the floating liquid was absorbed using the filter paper. Then, the copper mesh was added with 10 µL uranyl acetate (Xinxingbairui Technology, Beijing, China) for re-staining. After 1 min, the floating liquid was absorbed using the filter paper. After drying at room temperature, the samples were observed using a transmission electron microscope (TEM) (HT-7700, Hitachi, Tokyo, Japan), imaged at 100 kV, and photographed. The concentration of exosome particles was detected using Nanosight Tracking Analysis of ZetaView PMX 110 (Particle Metrix, Meerbusch, Germany), and the samples were diluted to make the diluted sample concentration between 10^8^/mL and 10^9^/mL.

### 2.5 ELISA

EGFR level was determined using the ELISA method following the ELISA kit (EH0010, FineTest, Wuhan, Hubei, China) instructions. The optical density value was read on a microplate reader (Bio-Rad 680; Bio-Rad, Hercules, CA, USA).

### 2.6 Chemiluminescence immunoassay

The levels of CA125, CEA, and CYFRA21-1 in the serum of patients were examined using an automatic electrochemiluminescence immunoanalyzer (ROCHE Modular Analytics E170, USA) and the supporting kit, which was all provided by Roche company (Switzerland).

### 2.7 RT-qPCR

The total RNA was extracted from peripheral blood and serum exosome samples using the TRIzol reagent (R&D SYSTEMS, Inc., MN, USA). cDNA was synthesized using the RT kit (Takara, Tokyo, Japan) with 1 µg RNA as a template. With cDNA as the template, the gene fragments were amplified. The qPCR reaction system (20 μL) included 2 μL cDNA template, 10 μL SYBR Premix ExTaq II (Takara), 1 μL upstream and downstream primers, and ddH_2_O was added till the samples were up to 20 μL. The reaction conditions were 94°C, 10 s, and 40 cycles of 95°C, 5 s, 55°C, 35 s, 72°C, 40 s, and final extending at 4°C for 10 min. With U6 as an internal reference, the relative expression was calculated according to the 2^−ΔΔCt^ method [18]. All RT-qPCR data were normalized by U6 for quantitative comparison. The RT-qPCR primers were all synthesized by Sangon Biotech (Shanghai, China). The RT-qPCR primers are listed in Table 1.

**Table 1:** Primer sequence

Name of primer	Sequences (5′–3′)
miR-27b F	GGCAAGCGCACCGAAGA
miR-27b R	AGTGCAGGGTCCGAGGTATT
U6 F	GGAGACACGCAAACGGAAG
U6 R	AGTGCAGGGTCCGAGGTATT

### 2.8 Statistical analysis

SPSS 21.0 (IBM Corp., Armonk, NY, USA) and GraphPad Prism 6.0 software (GraphPad Software, San Diego, CA, USA) were adopted for statistical analysis and mapping. Shapiro–Wilk test was applied for checking the normal distribution, and the data were expressed as mean ± standard deviation. An independent sample *t*-test was applied for comparisons among groups. The counting data were expressed as the case and percentage, and the Chi-square test was employed for comparisons among groups. The receiver operating characteristic (ROC) curve was plotted to evaluate the diagnostic efficiency of parameters and obtain the cutoff value. Pearson method was utilized to analyze the correlation between miR-27b levels in serum exosomes of NSCLC and CA125, CEA, CYFRA21-1, and EGFR. *P* < 0.05 was indicative of statistical significance.

## 3 Results

### 3.1 Comparative analysis of clinical data of enrolled subjects

There were no significant differences in age, sex ratio, the proportion of smokers, hemoglobin level, and serum calcium concentration between NSCLC and healthy subjects (all *P* > 0.05). According to the pathological stage, patients with NSCLC were assigned to the relatively early NSCLC group (NSCLC-early group, *N* = 64) and relatively advance NSCLC group (NSCLS-terminal group, *N* = 73). There was no apparent difference in pathological types between the two groups (*P* > 0.05). Compared with the control group, the serum levels of ALP and CA125, CEA and CYFRA21-1 were elevated in NSCLC-early/NSCLS-terminal groups, and the levels in the NSCLS-terminal group were further raised in comparison to the NSCLC-early group (*P* < 0.05) (Table 2).

**Table 2:** Comparison of clinical baseline characteristics

		Control	NSCLC-early	NSCLS-terminal	*X* ^2^	*P*
Number of cases (case)		60	64	73	—	—
Age (years)		59.4 ± 6.8	60.7 ± 5.2	61.3 ± 5.5	—	0.1703
Male (case)		35	38	42	0.04762	0.9765
Smoking (case)		33	38	46	0.8469	0.645
Pathology type	Squamous cell carcinoma	—	47	54	0.5021	0.778
	Adenocarcinoma	—	15	18		
	Large cell carcinoma	—	2	1		
Hb (g/L)		119.62 ± 18.73	122.45 ± 17.58	118.13 ± 19.25	—	0.3923
Calcium (mmol/L)		2.21 ± 0.25	2.24 ± 0.22	2.19 ± 0.27	—	0.5009
ALP (U/L)		65.5 ± 16.2	89.1 ± 21.7	128.3 ± 42.3	—	<0.0001
CA125 (U/mL)		4.08 ± 1.27	17.86 ± 8.31	35.54 ± 18.16		<0.0001
CEA (ng/mL)		1.78 ± 1.04	14.33 ± 9.64	33.09 ± 13.52		<0.0001
CYFRA21-1 (ng/mL)		1.94 ± 0.37	5.26 ± 2.34	9.80 ± 3.15		<0.0001

Notes: The counting data were expressed by the number of use cases; the Chi-square test was employed for the comparison among groups; the measurement data were expressed by quartile; Mann–Whitney U test was applied for comparison among groups. HB: hemoglobin; calcium: serum calcium; ALP: alkaline phosphatase; CEA: carcinoembryonic antigen; CA125: carcinoembryonic antigen 125; CYFRA21-1: cytokeratin 19 soluble fragment.

### 3.2 Identification of serum exosomes

**Figure 1:**
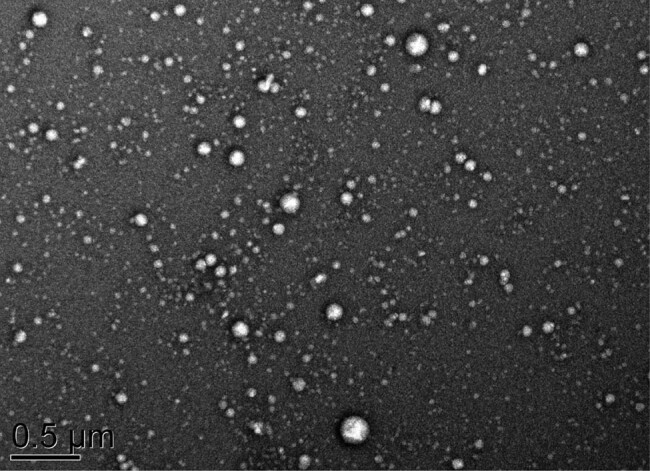
Morphology of exosome under TEM (uranyl acetate staining): Bar = 500 nm.

The exosomes in the serum of all subjects were extracted, and exosome samples after staining were observed using a TEM. TEM observed that the surface of exosomes was concave, showing a typical cup-shaped and lipid bilayer membrane bladder structure, with uniform size and a diameter of about 100 nm (Figure 1).

### 3.3 miR-27b was diminished in serum exosomes of the NSCLC patients

**Figure 2:**
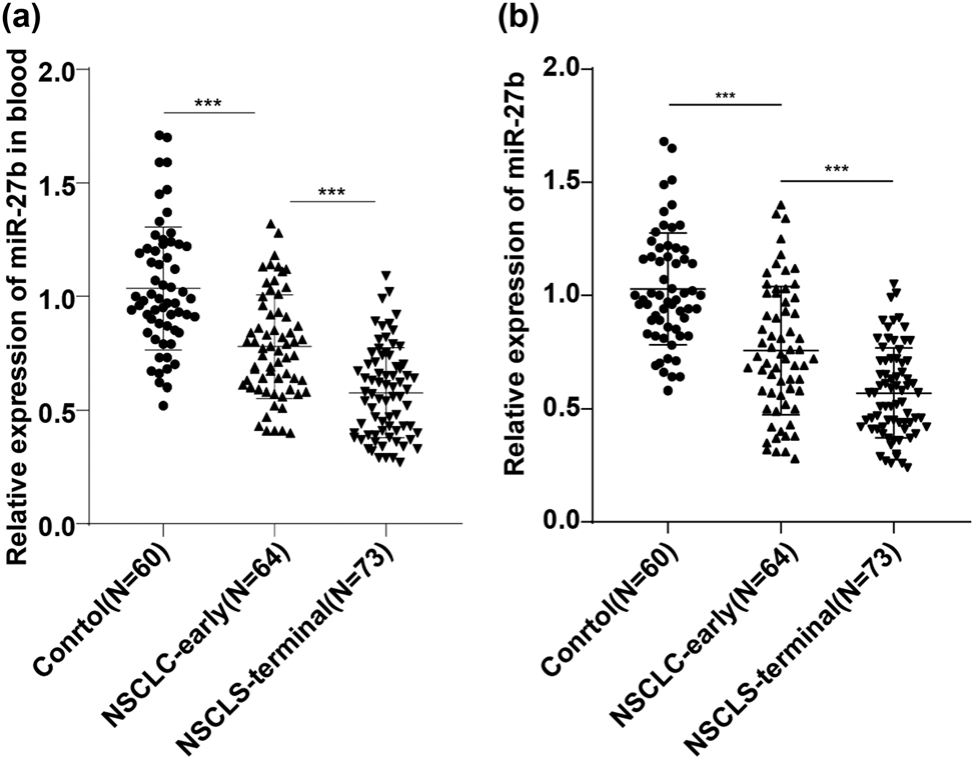
The expression of miR-27b was diminished in serum exosomes of NSCLC patients. (a) The expression of serum miR-27b was detected using RT-qPCR. (b) The expression of serum exosomal miR-27b was detected using RT-qPCR. The data were expressed as mean ± standard deviation. One-way analysis of variance (ANOVA) was applied for comparison among groups, followed by Tukey’s multiple comparisons test. ***P < 0.001.

The expressions of miR-27b in peripheral blood circulation and serum exosomes of healthy subjects and NSCLC patients were compared using RT-qPCR. Compared with the control group, miR-27b expression in peripheral blood circulation of the NSCLC group was suppressed, while the expression was further repressed in the NSCLS-terminal group relative to that in the NSCLC-early group (all *P* < 0.05) (Figure 2a); similarly, the expression of miR-27b in serum exosomes of NSCLC patients was also lower than that of the healthy people (Figure 2b). These results indicated that the expression trend of miR-27b in serum exosomes of patients with NSCLC was consistent with that in peripheral blood circulation.

### 3.4 Clinical diagnostic efficacy of miR-27b in NSCLC

**Figure 3:**
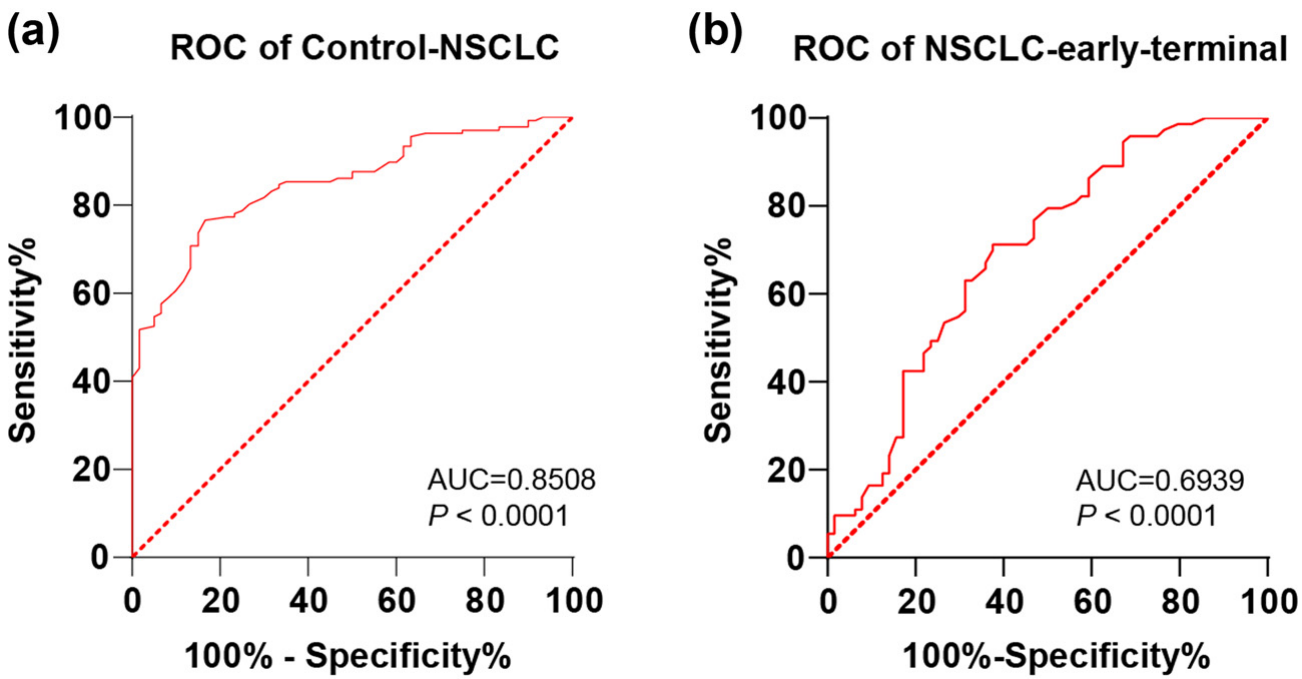
The ROC curve of miR-27b expression for NSCLC diagnosis: (a) The ROC curve of miR-27b for control and NSCLC diagnosis; (b) the ROC curve of miR-27b for relative NSCLC early/terminal; and the ROC analysis was employed for data analysis in panels a and b.

Considering the specific expression of miR-27b in serum exosomes of NSCLC patients, the ROC curve was further drawn to distinguish healthy subjects, relatively early NSCLC, and relatively advanced NSCLC according to miR-27b expression. The difference in diagnostic efficacy of miR-27b expression in the control group, NSCLC-early group, and NSCLC-terminal group was compared and analyzed using the MedCalc-Comparison of ROC curves. ROC analysis of the control group and NSCLC group revealed that the area under the curve (AUC) was 0.8508 (*P* < 0.0001, Figure 3a); the cut-off value was 0.8150, with 76.64% sensitivity and 83.33% specificity. ROC analysis of the NSCLC-early group and NSCLC-terminal group elicited that the AUC was 0.6939 (*P* < 0.0001, Figure 3b); the relatively advanced stage of NSCLC was judged by miR-27b < 0.6650, with 71.23% sensitivity and 62.50% specificity. These results manifested that miR-27b had certain clinical diagnostic efficacy for NSCLC.

### 3.5 Correlation between miR-27b and NSCLC indexes

**Figure 4:**
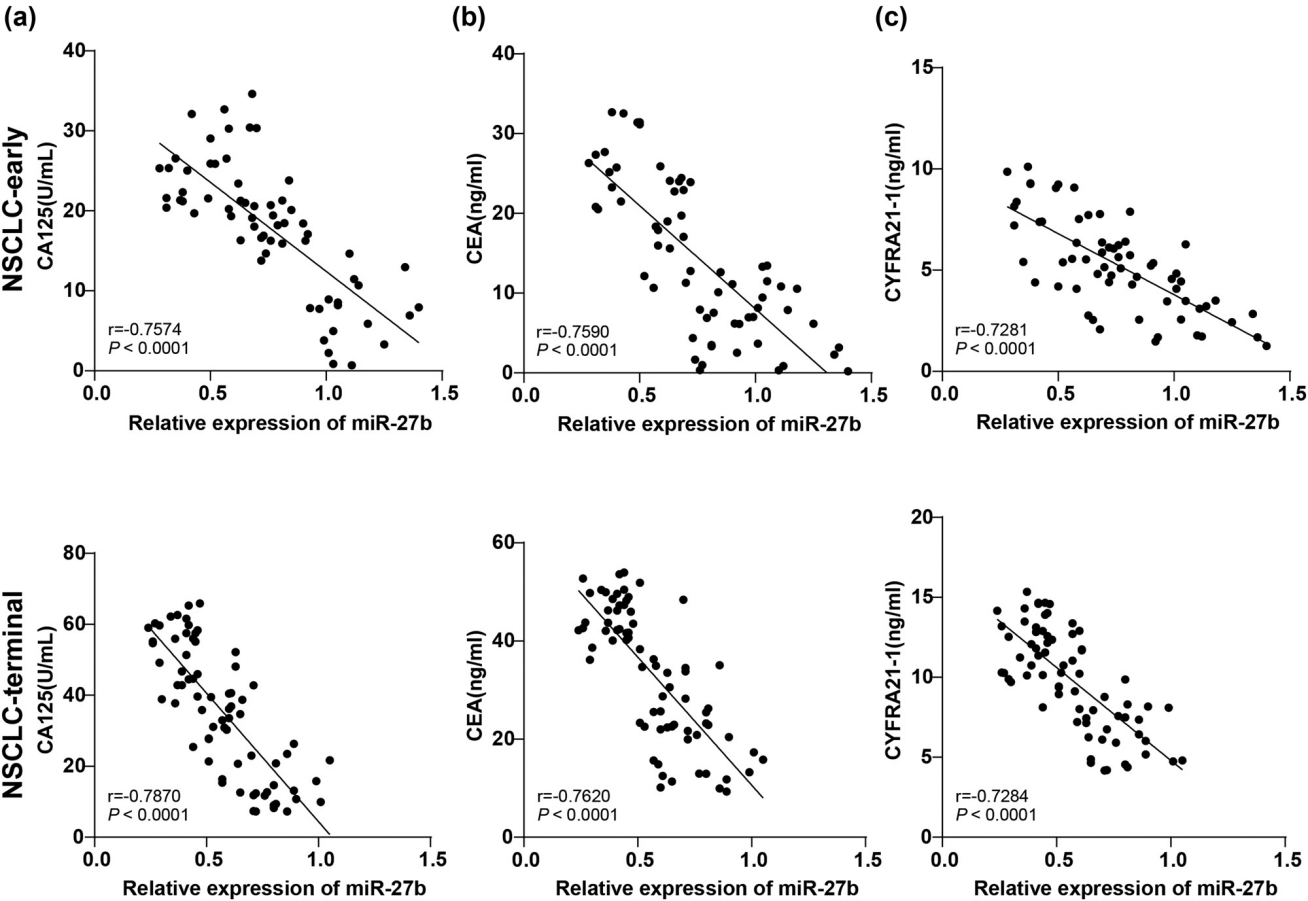
The correlation analysis between serum exosomal miR-27b and CA125, CEA, and CYFRA21-1 levels. (a) Pearson analysis was applied for correlation analysis between serum exosomal miR-27b expression in the NSCLC-early/NSCLC-terminal groups and CA125 level; (b) Pearson analysis was applied for correlation analysis between serum exosomal miR-27b expression in the NSCLC-early/NSCLC-terminal groups and CEA level; (c) Pearson analysis was applied for correlation analysis between serum exosomal miR-27b expression in the NSCLC-early/NSCLC-terminal groups and CYFRA21-1 level. The Pearson analysis was employed for data in panels a, b, and c.

The correlation between miR-27b expression in the NSCLC-early group and the NSCLC-terminal group and the levels of tumor markers CA125, CEA, and CYFRA21-1 was further analyzed. The levels of CA125, CEA, and CYFRA21-1 in serum exosomes of the NSCLC-early group and NSCLC-terminal group were negatively correlated with miR-27b expression (all *P <* 0.001, Figure 4).

### 3.6 EGFR level was raised in the serum of NSCLC patients and was negatively correlated with miR-27b

**Figure 5:**
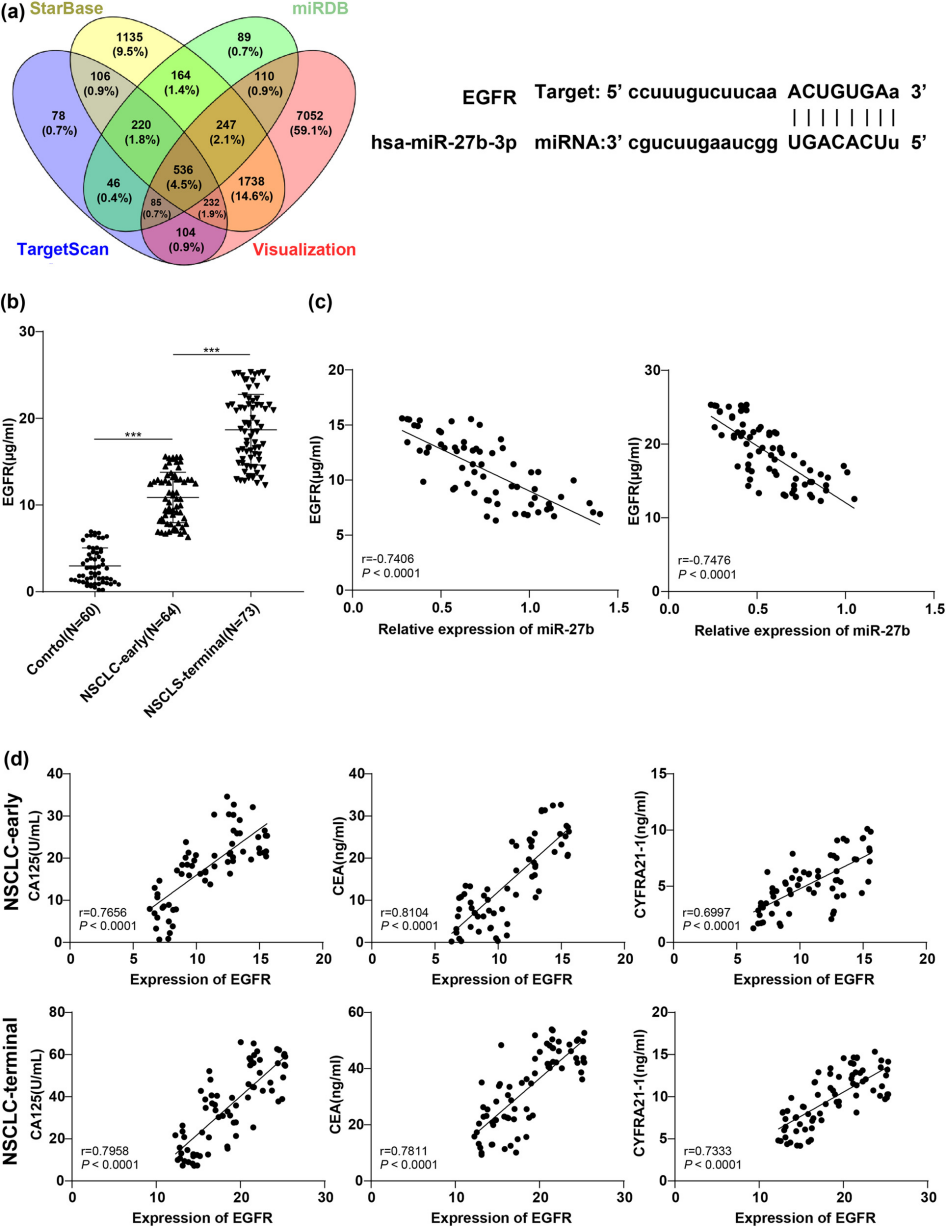
miR-27b might affect NSCLC progression through EGFR. (a) The target binding sites between miR-27b and EGFR were predicted and determined using a database; (b) the level of EGFR was detected by ELISA; (c) the correlation between miR-27b and EGFR level in the serum was analyzed using the Pearson analysis; (d) Pearson analysis was applied for correlation analysis between serum EGFR level in the NSCLC-early/NSCLC-terminal groups and CA125, CEA, and CYFRA21-1 levels. One-way analysis of variance (ANOVA) was applied for comparison among groups in panel b, followed by Tukey’s multiple comparisons test. ***P < 0.001. Pearson analysis was used for data analysis in panels c and d.

The downstream targets of miR-27b were predicted and screened using various miRNA target gene prediction databases ENCORI (http://starbase.sysu.edu.cn/), TargetScan (http://www.targetscan.org/vert_71/), miRDB (http://mirdb.org/), and miRNA Target Visualization (https://cm.jefferson.edu/rna22/Precomputed/). Among these, EGFR was identified. EGFR is a glycoprotein receptor on the surface of the cell membrane with tyrosine kinase activity and is the expression product of proto-oncogene Cerb-1; high expression of EGFR can promote tumor angiogenesis and the proliferation, adhesion, and invasion of tumor cells [19]. There were targeted binding sites between miR-27b and the 3′-UTR region of EGFR predicted using the Starbase website (Figure 5a). Therefore, we speculated that miR-27b might affect NSCLC through EGFR. The serum level of EGFR was detected using ELISA. Compared with the control group, the serum level of EGFR in NSCLC patients was stimulated, and the level in the NSCLC-terminal group was further facilitated relative to that in the NSCLC-early group (*P* < 0.001, Figure 5b). Furthermore, the correlation between miR-27b and EGFR level in the serum was analyzed using the Pearson analysis, which showed a negative correlation between miR-27b and EGFR (Figure 5c). Meanwhile, the correlation between EGFR level and CA125, CEA, and CYFRA21-1 was analyzed using Pearson analysis. EGFR was also positively correlated with the levels of serum tumor markers (*P* < 0.001, Figure 5d). Collectively, miR-27b in serum exosomes of NSCLC patients might affect NSCLC progression by affecting EGFR levels.

## 4 Discussion

Lung cancer is the most prevailing malignancy causing a huge amount of cancer death across the world, about 1.6 million deaths each year [2]. NSCLC is estimated to account for 85% of the total lung cancer [20]. Evidence has elucidated miR-27b as a potential biomarker for NSCLC [21]. This study illustrated that low expression of miR-27b assisted NSCLC diagnosis and miR-27b exerted effects on NSCLC by affecting the EGFR level.

It is reported that miR-27b is involved in the process in different tumors, such as colorectal cancer, lung cancer, and gastric cancer [16,22,23]. miR-27b is involved in the progression and development of NSCLC [24]. Our results demonstrated that expression of miR-27b in the peripheral blood circulation of NSCLC patients was repressed, while miR-27b was further repressed in advanced NSCLS patients relative to that in early NSCLC patients. Similarly, miR-27b expression in serum exosomes of NSCLC patients was also lower than that of healthy people. Consistently, miR-27b is diminished in NSCLC cell lines [24]. Briefly, miR-27b was downregulated in serum exosomes of the NSCLC patients. To analyze the diagnostic efficiency of miR-27b in NSCLC, we drew ROC curves of miR-27b for distinguishing healthy subjects and NSCLC patients in relatively early stage, and NSCLC patients in relatively advanced stage and found that miR-27b < 0.8150 could distinguish healthy subjects and NSCLC patients; miR-27b < 0.6650 could distinguish NSCLC patients in relatively early stage and NSCLC patients in the relatively advanced stage. Consistently, serum miRNAs, including miR-27b, may become cost-effective, sensitive biomarkers for the early diagnosis of NSCLC [25]. Briefly, miR-27b had certain clinical diagnostic efficacy for NSCLC.

CA125, CEA, and CYFRA21-1 are tumor markers [26]. Our results discovered that ALP and CA125, CEA, and CYFRA21-1 levels were raised in the serum of NSCLC patients and further enhanced in NSCLC patients in relatively advanced stage relative to NSCLC patients in the early stage. Consistently, ALP level is higher in lung cancer [27], and CA125, CEA, and CYFRA21-1 levels are elevated in NSCLC patients and are considered negative prognostic factors for both early and advanced stages of NSCLC [28]. Furthermore, we analyzed the correlation between miR-27b and the levels of these tumor markers and discovered that CA125, CEA, and CYFRA21-1 levels in NSCLC patients in early and advanced stages were negatively correlated with miR-27b expression. CYFRA21-1, CEA, and CA125 levels are associated with ocular metastases of lung cancer and become independent risk factors [29]. These results further proved the diagnostic value of miR-27b in NSCLC from the side.

To further study the downstream mechanism of miR-27b, we predicted the targets of miR-27b using various miRNA target gene prediction databases and identified EGFR. Many studies have shown that 90% of the NSCLC cases are characterized by overexpression and/or aberrant activation of EGFR [30,31], which is a cell surface receptor tyrosine kinase to which growth factors selectively bind. Upon ligand binding, EGFR undergoes homo- or heterodimerization with other EGFR family receptors, which leads to the autophosphorylation of mitogen-activated protein kinase, a signal transducer and activator of transcription 3, and mammalian target of rapamycin (mTOR). As a result, EGFR, once triggered, acts as the initiator of the signal transduction process, stimulating cell growth, and cell migration [32,33,34,35]. In addition, EGFR overexpression, and/or mutations within the tyrosine kinase domain, lead to an overactivation of the downstream signaling pathways, playing an important role in epithelial cell transformation. It has been reported that the inhibition of EGFR phosphorylation in A549 cells, overexpressing wild-type EGFR, results in the inhibition of mTOR, which is a key intracellular kinase involved in the regulation of proliferation and cell survival [36,37,38], thus playing a role in NSCLC. Our results identified that serum EGFR level in NSCLC patients was facilitated and further higher in NSCLC patients in relatively advanced stage than that of NSCLC patients in relatively early stage. Consistently, EGFR is frequently mutated and overexpressed in NSCLC [39]. Additionally, we studied the correlations of EGFR level with miR-27b and serum CA125, CEA, and CYFRA21-1, and noted that EGFR level was negatively correlated with miR-27b and positively correlated with serum tumor markers. Consistently, serum CEA level is related to EGFR gene mutation in NSCLC patients [40]. In conclusion, miR-27b in serum exosomes of NSCLC patients might affect NSCLC progression by affecting EGFR level.

In summary, as a prospective study, this study verified the miR-27b expression in serum exosomes of NSCLC patients and its clinical diagnostic value for the first time and provided a new entry point for the clinical judgment of NSCLC. However, the predictive value of miR-27b for NSCLC was only assessed by the miR-27b expression in serum exosomes, and the number of cases and events included was small. Further investigation is needed to expand the sample size, clarify the diagnostic ability of miR-27b, increase the reliability of the results, and redetermine the miR-27b expression in serum exosomes after treatment to study its predictive value in the prognosis of NSCLC patients. Moreover, this study only explored the correlation between exosomal miR-27b and serum EGFR level, indicating that miR-27b might affect the process of NSCLC by affecting the level of EGFR, but its specific effects and mechanism still needed to be further verified by more experiments. In the future, we will further explore the mechanism of miR-27b regulating EGFR in NSCLC. In addition, this study only used U6 as the internal reference to compare and analyze the relative expression of exosomal miR-27b, while Chevillet et al. observed that only a small minority (<3%) of cancer-associated biomarker miRNAs were recovered with classical exosomes isolated using gold standard differential ultracentrifugation-based methods. Although this result does not directly address the diagnostic use of these exosomes, it indicates that the majority of these established biomarkers are present in plasma and serum in other physical forms [41]. Therefore, more rigorous research is needed to detect the absolute quantification of miRNA in exosomes to determine the diagnostic use of classical exosomes. In addition, referring to previous studies [42,43,44,45], we selected U6 as the internal reference of RT-qPCR for the detection of miR-27b. During the experiment of quantitative circulating miRNAs, the changes in starting materials, sample collection, RNA extraction, and enzyme efficiency may introduce potential deviations and lead to quantitative errors. Considering these problems, developing an effective standardization strategy is very important to evaluate circulating miRNAs. Among the existing normalization methods, normalization for one stable internal reference gene (or better, a group of multiple stable internal reference genes) is the most accurate and appropriate method to evaluate circulating miRNAs [46]. It is reported that U6 may not be a widely used internal reference [47], unlike the assessment of cellular miRNA levels for which there are accepted housekeeping genes, analogous reference controls for normalization of circulating miRNAs in plasma and serum are lacking. The selection of internal miRNA control can improve the evaluation of disease-related changes in circulating miRNAs [48]. For example, the combination of miRNAs let-7d, let-7g, and let-7i has been reported to have highly consistent results in many healthy control groups and disease patients. These miRNAs are statistically superior to the most commonly used internal reference genes [49] in the quantification of serum miRNAs. We have not performed the quantitative detection of miR-27b expression in exosomes. In the future, we will pay more attention to the selection of internal parameters in the quantitative detection of serum exosomal miRNAs.
